# Overlapping Autoimmune Neurological Syndrome: A Case Report of Triple-Positive Antibody

**DOI:** 10.7759/cureus.29379

**Published:** 2022-09-20

**Authors:** Kyle Bonner, Hassan Aboul Nour, Anza B Memon

**Affiliations:** 1 Neurology, Wayne State University School of Medicine, Detroit, USA; 2 Neurology, Henry Ford Health System, Detroit, USA

**Keywords:** anti-nmda receptor encephalitis, autoimmune encephalitis, anti-achr antibody, aquaporin-4 (aqp4) antibody, neuro-immunology, neuromyelitis optica spectrum disorder (nmosd), myasthenia gravis (mg)

## Abstract

The presentation of several autoimmune neurological disorders in a single patient is rare and often debilitating. However, early diagnosis and efficacious treatment can lead to a significant recovery. Here, we present an interesting case of a triple antibody-positive autoimmune neurological syndrome patient who manifested the clinical features of neuromyelitis optica (NMO) spectrum disorder (NMOSD), N-methyl-D-aspartate (NMDA) receptor (NMDAR) encephalitis, and myasthenia gravis (MG). Hence, the patient manifested both central and peripheral nervous system immune-mediated neurological syndromes. A middle-aged female with a history of seropositive aquaporin-4 (AQP4) NMOSD on mycophenolate 1 g twice daily presented with severe fatigue and right eye ptosis (three months since NMOSD diagnosis) and tested positive for acetylcholine receptor (AchR) binding antibody, consistent with MG. Six months after the patient’s NMOSD diagnosis, she began to experience subacute progressive cognitive decline, behavioral changes, imbalance, anxiety/panic attacks, and paranoid delusions. NMDAR encephalitis was suspected, and she tested positive for cerebrospinal fluid NMDAR antibodies. After treatment with steroids failed, she was given two doses of rituximab 1 g, two weeks apart, and reported improvement in her symptoms shortly after the second dose.

## Introduction

The co-existence of several autoimmune conditions has been reported with neuromyelitis optica (NMO) spectrum disorders (NMOSD) [1]. While a rare disorder, there have been documented cases of concurrent N-methyl-D-aspartate (NMDA) encephalitis and anti-aquaporin-4 (AQP4) NMOSD [2,3]. In a case series of 691 patients with anti-NMDA receptor (NMDAR) encephalitis, 23 patients were identified with overlapping demyelinating syndromes associated with AQP4 and myelin oligodendrocyte glycoprotein (MOG) with clinical and radiographic features preceded or followed by the demyelinating disease process [3]. Another prospective observational study with the co-existence of MOG and AQP4 antibodies with anti-NMDAR showed that 0.6% of patients with NMOSD had overlapping NMDAR encephalitis and 11.9% of patients had anti-MOG disease [2]. The co-existence of myasthenia gravis (MG) has been reported with NMOSD [1].

In this case report, we present a middle-aged patient displaying the clinical manifestations of these three rare neurological disorders (NMOSD, MG, and anti-NMDAR encephalitis). By presenting this unusual case, we hope to help clinicians be more vigilant about the possibility of overlapping neurological syndromes that will help avoid delays in the diagnosis and treatment of these conditions. This article was previously presented as a meeting abstract at the 2022 American Academy of Neurology (AAN) Annual Meeting on April 3, 2022.

## Case presentation

**Figure 1:**
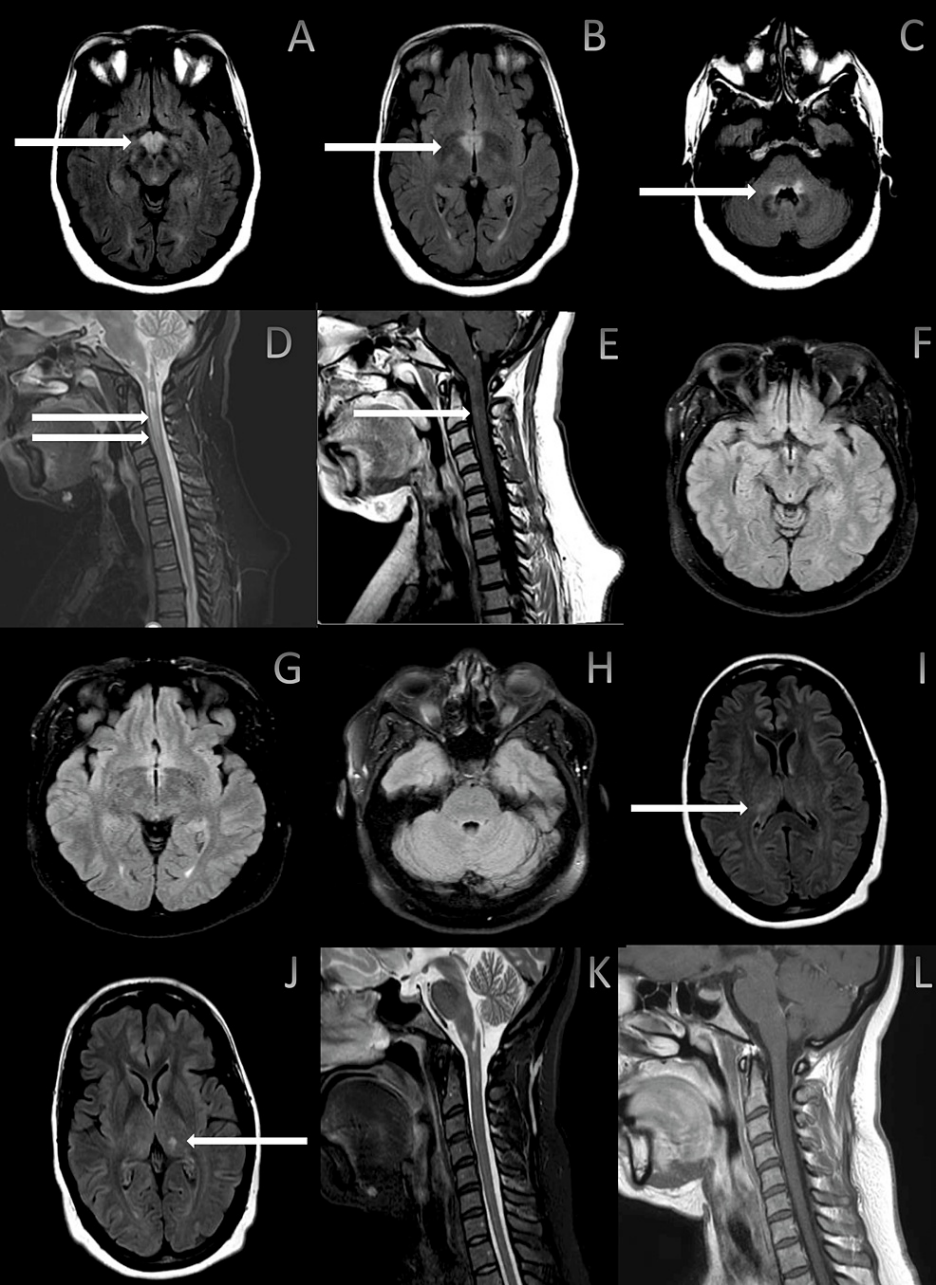
Magnetic resonance imaging (MRI) of the brain and cervical spine MRI of the brain fluid-attenuated inversion recovery axially showing T2 hyperintensities in the bilateral hypothalamic regions (A, B) and pontomedullary junction (C), with interval resolution imaging after a year (F-H). MRI of the cervical spine showing T2 signal changes from cervicomedullary junction to C3 level (D) with faint gadolinium enhancement (E). Repeat MRI of the brain, interval of two years, showing new T2 hyperintensities in the bilateral thalami (I, J). Repeat MRI of the cervical spine, interval of 18 months, showing improved T2 hyperintensities within cervical medullary junction (K), without enhancement (L)

A 44-year-old previously healthy female presented to an outside hospital emergency department with a headache followed by imbalance, right upper and lower extremity numbness, and weakness. Her examination findings on admission were notable for 3/5 strength of her right upper and lower extremities. She also had a loss of sensation in her right upper extremity. The rest of her examination was unremarkable. She had a head computed tomography (CT) performed, which was unremarkable. Magnetic resonance imaging (MRI) of the brain showed T2 hyperintensities in the bilateral hypothalamic regions and pontomedullary junction, prominent on the left side without associated diffusion restriction and enhancement (Figure 1A-1C). The patient refused a lumbar puncture and was told to follow up with a neurology outpatient. At her three-month follow-up, she reported that her symptoms have gradually improved with physical therapy and now only has residual right-handed numbness. As a demyelinating process was suspected, she had an extensive neurological workup over the next three months. She underwent a lumbar puncture with an opening pressure of 12 cm H2O, a protein count of 35 mg/dl, and a cell count of 1/mm^3^. Serum studies showed negative cell-based assay serology for AQP4 immunoglobulin (Ig) G and MOG-IgG and mildly elevated ribonuclear protein (RNP) and Sjogren’s antibody for soluble substance A/Ro (SS-Ro) (Table 1). Workup for sarcoidosis was negative with normal angiotensin-converting enzyme levels and normal chest CT without mediastinal and hilar lymphadenopathy. MRI of the cervical spine revealed T2 signal changes from the cervicomedullary junction to C3 level with faint enhancement (Figure 1D, 1E). MRI of the thoracic spine was normal without signal changes. Cerebrospinal fluid analysis and paraneoplastic panel were negative except for mildly elevated N-type calcium channel antibodies of 0.04 and mildly elevated IgG index of 0.8 (Table 2).

**Table 1:** Patient initial serum laboratory results with reference values Ab: antibody; AchR: acetylcholine receptor; AGNA-1: anti-glial nuclear antibody type 1; ANA: antinuclear antibody; ANCA: antineutrophil cytoplasmic antibodies; ANNA: antineuronal nuclear antibody; C-ANCA: antineutrophil cytoplasmic autoantibody, cytoplasmic; CRMP-5: collapsin response-mediator protein-5; ds: double-stranded; DRVVT: dilute Russell viper venom time; GAD: glutamic acid decarboxylase; HB: hepatitis B; Ig: immunoglobulin; P-ANCA: perinuclear anti-neutrophil cytoplasmic antibodies; PCA: Purkinje cytoplasmic antibody; PCA-Tr: Purkinje cytoplasmic antibody titer; PT: prothrombin time; PTT: partial thromboplastin time; S: serum; SM: smooth muscle; SS: Sjogren’s syndrome; TPMT: thiopurine S-methyltransferase; ELISA: enzyme-linked immunosorbent assay; HBsAg: hepatitis B surface antigen; HBc: hepatitis B core; IFA: immunofluorescent assay

Laboratories	Values	Reference range and units
ANA screen and titer	Negative	Negative, <1:80 titer
*Borrelia burgdorferi* total IgG/IgM Ab	0.23	<0.90 index value
C-reactive protein	0.1	<0.5 mg/dl
Erythrocyte sedimentation rate	19	<20 mm/hour
HBsAg	Negative	Negative
HBc Ab IgM	Negative	Negative
Hepatitis C Ab	Negative	Negative
Hepatitis A Ab IgM	Negative	Negative
International normalized ratio	0.94	1
Myelin oligodendrocyte glycoprotein-IgG1	Negative	Negative
Neuromyelitis optica/aquaporin-4 IgG	Negative	Negative
N-Methyl-D-aspartate receptor Ab, IgG	<1:10	<1:10
Acetylcholine receptorAb	0.53	<0.3 nmo/L
B2 glycoprotein IgG Ab	<9	<20 SGU
B2 glycoprotein IgM Ab	<9	<20 SMU
B2 glycoprotein IgA Ab	<9	<20 SMU
Angiotensin-converting enzyme	39	8-52 U/L
Methylmalonic acid	0.13	<0.40 umol/L
Quantiferon TB Plus	Negative	Negative
IgG	1939	700-1600 mg/dl
Albumin	3963	3848-5304 mg/dl
Ribonuclear protein Ab	1.8	<1.0 ELISA units
SM Ab	0.2	<1.0 ELISA units
SS A/Ro Ab	3.0	<1.0 ELISA units
SS B/La Ab	<0.2	<1.0 ELISA units
Treponemal IgG/IgM	Nonreactive	Nonreactive
C-ANCA	<1:20	<1:20 titer
P-ANCA	<1:20	<1:20 titer
PT	12.7	12.1-14.5 seconds
PTT	34	22-36 seconds
DRVVT	31	27-45 seconds
Lupus anticoagulant	37.5 seconds	30.3-43.2 seconds
DNA Ab (ds) *Crithidia*, IFA	Negative	Negative
TPMT enzyme activity	26.2	>21 EU
Cardiolipin Ab IgA	42.9	<12 APL
Cardiolipin Ab IgG	<9.0	<15 GPL
Cardiolipin Ab IgM	<9.0	<12.5 MPL
Vitamin B1, whole blood	50	38-122 ug/L
Varicella IgG Ab	2.6	<1.0 units
Varicella IgM Ab	1.26	<0.9 index
Striated muscle Ab IgG	1:40	<1:40
AchR ganglionic neuronal Ab, S	0.00	<0.02 nmol/L
Amphiphysin Ab, S	Negative	<1:240 titer
AGNA-1, S	Negative	<1:240 titer
ANNA-1, S	Negative	<1:240 titer
ANNA-2, S	Negative	<1:240 titer
ANNA-3, S	Negative	<1:240 titer
CRMP-5-IgG, S	Negative	<1:240 titer
GAD65 Ab, S	0.00	0.02
Neuronal (V-G) K+ channel Ab, S	Negative	<0.02 nmol/L
N-type calcium channel Ab	0.04	<0.03 nmol/L
P/Q-type calcium channel Ab	0.00	<0.02 nmol/L
PCA-1, S	Negative	<1:240
PCA-2, S	Negative	<1:240
PCA-Tr, S	Negative	<1:240
Striational (striated muscle) Ab, S	Negative	<1:120

**Table 2:** Patient CSF initial and repeat (18 months later) laboratory results with reference values Ab: antibody; AGNA-1: anti-glial nuclear antibody type 1; ANNA: antineuronal nuclear antibody; CSF: cerebrospinal fluid; CRMP-5: collapsin response-mediator protein-5; EBV: Epstein-Barr virus; HSV: herpes simplex virus; Ig: immunoglobulin; PCA: Purkinje cytoplasmic antibody; PCA-Tr: Purkinje cytoplasmic antibody titer; PCR: polymerase chain reaction; S: serum; VDRL: venereal disease research laboratory

Laboratories	Initial values	Repeat (18 months) values	Reference range and units
N-Methyl-D-aspartate receptor Ab IgG serum with reflex to titer	-	1:5	<1:1
West Nile IgG Abs	-	<1.3	<1.30 antibody not detected, 1.30-1.49 equivocal, and >1.49 antibody detected
West Nile IgM Abs	-	<0.9	<0.90 antibody not detected, 0.90-1.10 equivocal, and >1.10 antibody detected
Varicella zoster, PCR	-	Not detected	Not detected
Cytomegalovirus PCR, qualitative	Not detected	Not detected	Not detected
EBV DNA, PCR CSF	-	Not detected	Not detected
HSV 1 DNA	-	Not detected	
HSV 2 DNA	-	Not detected	
VDRL	Nonreactive	Nonreactive	
Protein	34.9	50	15-55 mg/dl
Lactic acid	-	1.4	1.2-2.4 mmol/L
Glucose	64	58	40-80 mg/dl
Red blood cells	1	<3	0/mm^3^
White blood cells	1	7	0-5/mm^3^
Neutrophils	0	0	0%-6%
Lymphocytes	94	97	40%-80%
Monocytes	6	2	15%-45%
Mononucleates	0	0	15%-45%
Macrophages	0	0	0%
Eosinophils	0	0	0%
Basophils	0	0	0%
IgG	7.4	6.1	0.5-6.1 mg/dl
Albumin	18.7	18.7	10-31 mg/dl
IgG index/CSF	0.8	0.8	0.3-0.7 ratio
Angiotensin-converting enzyme	<5	-	<15 U/L
Amphiphysin Ab, S	Negative	-	<1:240 titer
AGNA-1, S	Negative	-	<1:240 titer
ANNA-1, S	Negative	-	<1:240 titer
ANNA-2, S	Negative	-	<1:240 titer
ANNA-3, S	Negative	-	<1:240 titer
CRMP-5-IgG, S	Negative	-	<1:240 titer
PCA-1, S	Negative	-	<1:240
PCA-2, S	Negative	-	<1:240
PCA-Tr, S	Negative	-	<1:240

The patient was recommended clinical observation and a repeat MRI of the brain and NMO IgG in six months. She had a repeat MRI of the brain a year later (delayed due to the COVID-19 pandemic), which showed complete resolution of the initially reported T2 signal changes in the bilateral hypothalamus and pontomedullary junction (Figure 1F-1H). Re-imaging of the cervical and thoracic spine was not performed at this time. NMO IgG serology was repeated and was positive with a titer of 1:100 one year after she initially tested negative (Table 1). To prevent future attacks, the patient was started on mycophenolate mofetil 1 g twice daily for AQP4-positive NMOSD. A few months later, the patient presented with severe fatigue, diplopia, anorexia, generalized weakness, and an unintentional 30 lb weight loss. Neurological examination was normal except for mild right fatigable ptosis. Laboratory testing showed normal complete blood count; comprehensive metabolic panel, thyroid profile, folate level, iron profile, and monoclonal protein evaluation; and low normal vitamin B12 level of 189 pg/ml with normal methylmalonic acid. She had normal glycated hemoglobin, parathyroid hormone, and vitamin D levels. Repeat antinuclear antigen was positive with a titer of 1:320 homogenous. Due to her constellation of symptoms, she was tested for acetylcholine receptor (AchR) binding antibodies, which were positive at 0.53 nmol/L, consistent with the diagnosis of MG. The patient declined neurophysiological testing at that time. She underwent a chest/abdomen/pelvis CT for evaluation of a thymoma, which was negative for malignancy. She was started on pyridostigmine 30 mg three times/day for MG-related fatigue and generalized weakness.

Eighteen months after her NMOSD diagnosis, the patient presented with subacute progressive cognitive decline, imbalance, and behavioral problems. She was accompanied by her sister, who reported that the patient was having severe panic attacks, bizarre behavior, paranoid delusions, and short-term memory problems. Neither the patient nor her sister reported a history of anxiety. Neurological examination showed mild cognitive impairment and impaired tandem gait. On the Montreal Cognitive Assessment, she scored 24/30 and showed difficulty with executive, visuospatial functioning, recall, and fluency. Given the subacute progressive cognitive decline, there was a high suspicion of encephalitis and vasculitis. A repeat MRI of the brain/cervical/thoracic spine was performed and showed new areas of T2 hyperintense foci within the bilateral posterior thalami without associated enhancement (Figure 1I, 1J). In the cervical spine, there was improvement in the previously reported T2 signal changes without new lesions (Figure 1K, 1L). The thoracic spine showed only mild degenerative changes and was negative for any demyelinating lesions.

Repeat lumbar puncture showed normal cell count, glucose, and protein (Table 2). Cerebrospinal fluid viral polymerase chain reactions were negative. Cerebrospinal fluid was positive for anti-NMDAR IgG at 1:5, indicative of NMDAR encephalitis. Serum anti-NMDAR IgG was negative (Table 1). She was given a three-day course of intravenous methylprednisolone 1 g without improvement. She had chest/abdomen/pelvis CT, which showed a right ovarian cyst but was negative for malignancy. Transvaginal ultrasound was performed and negative for a possible teratoma. The patient received rituximab 1 g, two doses, two weeks apart, and her anxiety, behavioral symptoms, and cognition significantly improved.

The patient was seen most recently eight months after her first rituximab infusion. She has received two rounds of infusions so far per her maintenance therapy plan. Her cognition and focal neurological deficits have remained stable. She has not had a relapse of her NMOSD or MG symptoms. She continues to suffer from anxiety and depression, which is being treated with clonazepam 0.5 mg at bedtime. Additionally, she reported a new sugar craving and weight gain, which is thought to possibly be related to limbic involvement of her NMDAR encephalitis. She was referred to behavior health for assistance in treating the symptoms, and the patient will follow up before each infusion (every six months).

## Discussion

While these three conditions are rare, there have been documented cases of concurrent NMOSD-NMDAR encephalitis and NMOSD-MG [3-8]. We could not find any literature related to a patient who exhibited the symptoms of these disorders while testing positive for all three antibodies (AQP4, AchR, and NMDAR) on different occasions.

The clinical significance of patients with overlapping NMOSD-NMDA encephalitis remains unclear. However, overlapping cases typically presented with more severe symptoms and required high-efficacy treatment [3]. In prior documented cases, there does not appear to be a pattern of one condition preceding the other. Patients who develop these disorders may test positive for only one antibody at the time of diagnosis and later develop detectable levels of the other antibodies [2,3]. It is thought that the immune upregulation involved in the pathogenesis of these syndromes may increase one’s susceptibility to other autoimmune disorders. These two conditions have very different clinical manifestations. Clinicians treating patients with NMOSD who later develop psychiatric symptoms, such as bizarre behavior, delusions, or hallucinations, should consider testing for anti-NMDAR.

While there are no published cases of patients with NMDA encephalitis and MG, there have been reports of concurrent MG and leucine-rich glioma-inactivated 1 (LGI1) protein antibody-associated encephalitis. This disease process presents with characteristic faciobrachial seizures and the same psychiatric symptoms as NMDA encephalitis [9]. The patient has not been tested for anti-LGI1 IgG at the time of writing. However, the patient’s lack of seizures and negative limbic involvement on imaging make LGI1 encephalitis unlikely. However, the association between these disorders should prompt anti-LGI1 IgG testing when patients with MG present with symptoms of encephalitis.

In patients with concurrent MG and anti-AQP4 NMOSD, MG typically presented several years before NMOSD, and the presentation of NMOSD did not correlate with cessation of MG immunosuppressive therapy [4,8,10]. Patients typically tested positive for the anti-AQP4 antibody at the time of MG presentation but did not show symptoms until years later [8]. Concurrent MG was not related to a worse prognosis in patients with AQP4-NMOSD [10]. While thymic hyperplasia is a well-known source of anti-AchR, new evidence suggests that the thymus could be a source of anti-AQP4 [7]. This could explain the increased incidence of these disorders occurring together compared to chance. Although the patient’s imaging was negative for a thymoma, CT is only 35% sensitive for thymic hyperplasia and 20% sensitive for a focal thymic mass [11]. Therefore, the thymic origin of these disorders cannot be excluded. Individuals diagnosed with MG should be tested for anti-AQP4 and counseled on the possibility of developing NMOSD in the future.

The subsequent manifestations of these rare disorders are likely a consequence of coexisting autoimmunity. NMDA encephalitis has been associated with concomitant demyelinating syndromes [3]. The mechanism behind this phenomenon remains unclear. Malignancies and their associated paraneoplastic syndromes often cause new-onset autoimmune disorders, including those seen in this patient. However, the patient’s workup has been negative for malignancy thus far.

Rituximab is a monoclonal antibody against the CD20 B-cell surface receptor. The binding of the antibody to this receptor triggers B-cell death and has a powerful systematic immunosuppressive effect [12,13]. Conveniently, rituximab has been shown to be an efficacious treatment for all three disorders [14-16]. A systematic review found that 30% of AchR+ patients saw decreased posttreatment antibody titers [14]. Response to rituximab is strong, with the majority of patients having some or all of their encephalitis symptoms reversed posttreatment [16]. We believe the benefit of treating these three conditions with a single drug will provide the best possible outcome for the patient with the least amount of side effects.

## Conclusions

Over the course of two years, this patient developed the clinical manifestations of three rare neurological autoimmune disorders at subsequent points in time. Not only did the symptoms of each disease overlap, but also the patient had positive serology for all three conditions simultaneously. After failing first-line therapy with glucocorticoids, she continues to respond well to rituximab. It is essential to address poor response to first-line immunosuppressive therapies early on, as the continuation of these therapies may not be effective in some cases. Additionally, central (NMOSD and NMDAR encephalitis) and peripheral (MG) neurological syndromes can occur simultaneously and even present at distant points in time. Clinicians need to be vigilant about the overlap of neurological syndromes affecting the central and peripheral nervous systems to avoid delays in the diagnosis and treatment to prevent poor clinical outcomes.
